# Supplementary material for: Are carbon emissions trading and green financial instruments synergistic? -Comprehensive quantitative research based on content analysis
Source: PLoS One. 2024 Mar 7;19(3):e0298601. doi: 10.1371/journal.pone.0298601 (PMC10919601; doi:10.1371/journal.pone.0298601)
Supplement: S1 Data — (ZIP) [file pone.0298601.s001.zip › Data/地级市Z_F工作报告.zip!/┴╔─■/╙¬┐┌╩╨/└┤╫╘╣1⁄2╓┌║┼í╛┬φ┐╦╩2╛▌═°í┐_2018.docx]

各位代表：

现在，我代表市政府向大会报告工作，请予审议，并请市政协委员提出意见。

一、过去五年及2017年工作回顾

本届政府履职的五年，是极不平凡的五年。面对经济发展进入新常态等一系列深刻变化，在市委的坚强领导下，市政府认真学习贯彻党的十九大精神，以习近平新时代中国特色社会主义思想为指导，全面落实“四个着力”“三个推进”要求，统筹推进“稳增长、促改革、调结构、惠民生、防风险”各项工作，全市经济发展稳中有进、社会各项事业全面进步。

五年来，我们始终坚持新发展理念，质量和效益同步提升。在做实经济数据的基础上，经济持续运行在合理区间。地区生产总值年均增长3.8%，居民消费价格年均上涨1.56%、城镇登记失业率控制在3.01%以内。财政收入质量不断提升，非税收入占比下降6个百分点，政府存量债务逐步化解，风险总体可控。三次产业比重调整到8.8:43.2:48，高新技术产品增加值、战略性新兴产业增加值占比分别达到25.6%、18%。

五年来，我们持续深化改革开放，新生动能逐步积聚。清理调整行政职权事项2205项，组建独立的行政审批局，行政审批效率提高80%。组建企业集团10家，在全省率先实现经营性国有资产集中统一监管。获批国家东北地区民营经济发展改革示范市、综合运输服务示范市等一批改革试点，民营经济增加值占比达到78.7%。进出口总额完成1971亿元，外贸依存度由25%上升至28.3%。辽宁自贸区营口片区、综合保税区（以下简称自贸区、综保区）获批成立，为振兴发展再添新引擎。深度融入“一带一路”，“营满欧”大陆桥建设取得突破性进展，集装箱海铁联运项目成为全国首批多式联运示范工程，联运量居全国首位。

五年来，我们统筹推进城乡建设，城市面貌持续改善。加快新型城镇化步伐，新增城区面积36.1平方公里，常住人口城镇化率达到64%以上。完成棚户区改造48567套、农村危房改造19797户。投入城建资金114亿元，东海大街、辽河护岸等一批重大基础设施投入使用。新建改造公路947公里，兰旗机场建成通航，去年旅客吞吐量突破20万人次，海陆空立体交通网络初步形成。建设美丽乡村示范村126个，农村环境得到改善。全面实施蓝天、碧水、青山、净土和农村环保“五大工程”，生态环境建设有效加强。森林覆盖率达到45%，城市污水处理率达到92%，我市获评国家森林城市、全国绿化模范城市。

五年来，我们着力保障和改善民生，人民群众获得感幸福感明显增强。投入民生和社会事业资金1041亿元，一大批热点、难点问题逐步解决。人民生活水平不断提高，城镇、农村常住居民人均可支配收入分别由23326元、10096元提高到34580元、15608元，分别增长48.2%、54.6%，稳定脱贫92921人。覆盖城乡的社会保障体系更加完善，保障水平不断提高，城乡低保标准分别由年人均5160元、2800元提高到6780元、4060元，分别增长31.4%、45%；农村特困人员集中供养、分散供养标准分别由年人均5050元、3525元提高到7163元、5063元，分别增长41.8%、43.6%；企业退休人员年人均养老金由19164元提高到25788元，增长34.6%。社会各项事业全面进步。六县（市）区全部通过义务教育发展基本均衡县（市）区国家验收，走在全省前列，营口理工学院获批成立，高等教育实现历史性突破。异地新建市中心血站、妇幼保健中心等一批医疗卫生基础设施，修建各类体育场所2800个，农村公益电影放映4.2万场。社会治理不断加强，刑事案件发案率下降6.4%，我市获评全省法治宣传教育先进市、平安辽宁建设先进市，实现全国双拥模范城“四连冠”。

五年来，我们严格落实全面从严治党要求，政府自身建设不断加强。扎实开展党的群众路线教育实践活动、“三严三实”专题教育、“两学一做”学习教育，把全面从严治党贯穿政府工作全过程，切实履行“一岗双责”，认真落实中央八项规定精神，驰而不息纠正“四风”，严查违规违纪问题，公务人员廉洁从政意识不断增强，政府自身建设取得新成效。

各位代表，2017年是本届政府收官之年，全市经济社会发展稳中向好。预计全市地区生产总值完成1270亿元，增长7%；固定资产投资464亿元，增长13%；一般公共预算收入113.7亿元，增长8.1%；城镇、农村常住居民人均可支配收入分别达到34580元、15608元，均增长7%左右。

（一）抓招商、上项目，不断增强经济发展后劲

招商引资和项目建设走在全省前列。制定出台产业招商重点指引、招商引资中介人奖励办法等一系列政策，有效落实项目和企业全周期“四个一”帮扶制度。全年开复工千万元以上项目539个，其中亿元以上项目259个。总投资超百亿元的忠旺集团、鲅鱼圈大白鲸海岸城等一批重大项目正在加快建设。有效盘活存量资源，清理处置闲置土地18.3公顷、厂房42.3万平方米，一批企业借助存量资源顺利落户开工。

城际交流合作实现新突破。以辽宁与江苏对口合作为契机，积极对接先进地区，与连云港等城市签订战略合作协议，与四平、温州、佛山、泉州等市缔结友好城市。与昆山合作共建的产业园区启动实施，与浙江海龟科技公司合作建设的国家级孵化器项目有序推进，开创了城际交流合作新模式。

营商环境持续改善。深入开展六大环境建设、九个专项行动，以“零容忍”的态度倒逼作风改进，处理问责132人，办结涉企投诉案件142件，招商引资政策不兑现等不讲诚信问题得到遏制。

（二）转方式、促调整，加快推动产业优化升级

创新驱动正在提速。全年转化科技成果50项，有效发明专利增长16%，新增国家级高新技术企业20户，我市被确定为国家知识产权试点城市。

现代农业稳步发展。农业增加值预计完成114亿元，增长3.8%。粮食产量实现66万吨，新建续建千万元以上农业产业化项目17个，新增农民专业合作社245家、家庭农场17个，完成农村土地确权登记任务24.9万亩。

工业经济快速回升。规模以上工业增加值预计完成330亿元，增长10%。支柱产业提速发展，企业效益明显好转，规模以上工业企业主营业务收入增长30%。实施重点技术创新项目108项，沈鼓压缩机组、金辰机械光伏生产线技术填补国内行业空白。完成“个转企”1144户，新增规模以上工业企业55户，新增产值超10亿元企业4户。

服务业稳中有进。服务业增加值预计完成610亿元，增长6.1%。新增物流企业900余户，以港口为依托的现代物流产业实现较快发展。全国首家企业社交商务平台（东北）腾讯企点产业中心正式落户，营口电商谷产业园投入使用，村级电商服务网点覆盖率达到87%。赤山旅游景区和辽宁团山国家海洋公园获批国家AAAA级旅游景区。全年旅游总收入235亿元，增长12.1%。引进金融机构5家，新增社会融资总量超500亿元，金辰机械主板上市，金融服务实体经济能力持续增强。

（三）建平台、强载体，持续扩大对外开放

沿海经济带建设再上台阶。编制三年攻坚计划，重点园区发展目标和产业定位进一步明晰，每个园区都明确了2—3个主导产业，实现差异化发展。加快人事制度、薪酬制度和管理体制改革，着力推动园区规范整合，北海新区、仙人岛能源化工区、中小企业创业园区晋升为省级开发区。

港口开放持续深化。全年港口货物吞吐量完成3.62亿吨，增长3%；集装箱运量627万标准箱，增长3%。中欧班列完成集装箱运量5.5万标准箱，增长30%；海铁联运量实现72.2万标准箱，增长37%。营口港成为全国沿海开通中欧班列数量最密集的港口。

自贸区建设实现良好开局。制度创新走在全省前列，复制推广上海等自贸区创新经验164条，省内首创“46证合一”制度和“16+X”集中监管执法模式。综保区建设顺利推进，国际邮件处理中心即将投入使用。全年自贸区新增注册企业2123户，610户实现运营。

（四）抓改革、破难题，有效激发经济发展活力

供给侧结构性改革收获新成果。扎实有效去产能、去库存、去杠杆，淘汰镁产业落后产能90万吨，销售商品房203万平方米，规模以上工业企业资产负债率下降2.1个百分点。多措并举降成本，为企业减负21.7亿元。突出重点补短板，13821人实现稳定脱贫、10个贫困村退出，投资近50亿元的24件民生实事基本完成年度计划任务。

“放管服”改革取得新成效。清理调整行政职权事项236项，网上审批平台上线运行，49项行政许可事项实现“不见面”审批。强化权责清单管理和动态调整，事中事后监管体系不断完善。城市执法体制改革取得新进展，住房城乡建设等6个领域城市管理行政执法职责实现集中行使。

国资国企改革实现新进展。积极配合开展辽宁港口整合，完成排水公司、医疗集团混合所有制改革和4家央企“三供一业”10个项目的分离移交工作。

事业单位改革迈出新步伐。按期完成44家生产经营性事业单位改革，精简归并事业单位115家，一次性收回事业编制6904个。在全国率先通过行政类事业单位改革试点市验收。组建营口新闻传媒中心和辽海文化传媒有限公司，实现新闻媒体融合发展。

（五）强建设、重治理，逐步优化城乡人居环境

城乡面貌进一步改观。完成市府路、盼盼路等道路改造，主干道路亮化、绿化水平持续提升。新建改建农村公路82.6公里，完成24个乡镇农村电网改造，对大中小河流、沟渠、库塘垃圾进行全面清理。柳树镇东岗子村获评国家环境整治示范村。

生态建设持续加强。拆除小锅炉557台，淘汰黄标车及老旧车辆13744台，城市空气质量逐步改善。有序推进北部污水处理厂建设，大辽河等主要河流水质进一步提升。扎实整改中央环保督察、国家海洋督察反馈问题，城市生活垃圾处理厂投入使用，永远角存量垃圾和城市污水处理厂存量污泥治理工程全面开工，一批突出环境问题得到有效解决。

新型城镇化扎实推进。坚持以特色乡镇建设引领城镇化发展，熊岳镇获批国家级特色小镇、红旗镇何家沟获批全国运动休闲特色小镇。

（六）补短板、兜底线，努力改善人民群众生活

就业和社会保障更加充分。实施积极就业政策，城镇新增就业1.98万人。强化养老保险基金征缴，多年断保欠费问题得到有效解决。企业退休人员养老金提高6.1%，城乡低保标准分别提高5.6%、7.7%，农村特困人员供养标准平均提高7%，城镇居民医保和新农合年人均财政补助标准提高30元。社区居家养老服务试点扎实推进。

社会各项事业协调发展。上下联动推进教育行风整治，教育质量不断提升，高考一本升学率提高4个百分点。引进辽宁方大集团开展医疗项目合作，全市医疗资源布局进一步优化。医药卫生体制改革取得新突破，公立医院全部取消药品加成。文体惠民工程深入实施，文化综合体项目启动，图书大厦投入使用。全面开展社区网格化服务管理，17个社区服务社会化试点启动实施。食品药品安全监管能力不断提升，人民群众饮食用药安全得到有效保障。

社会治理全面加强。开展“七五”普法，公共法律服务体系进一步完善。持续推进平安营口建设，立体化社会治安防控体系建设取得新成果。进一步畅通诉求渠道，一批积案难案得到妥善化解。安全生产网格化管理体系初步形成，全市安全生产形势总体平稳。

双拥共建、国防教育、民兵预备役和人民防空工作深入开展，国家安全、统计、民族、宗教、史志、档案、气象、地震等工作取得新进步；慈善、红十字、老龄、妇女儿童、残疾人等各项事业不断巩固提升。

（七）转作风、抓落实，切实提升政府执行力

坚持依法依规抓落实，坚决按照法定权限和程序行使权力，认真执行市人大及其常委会决议、决定，自觉接受市人大法律监督、工作监督和市政协民主监督，全年办理人大代表建议246件，政协提案279件，满意率99.4%。坚持强化责任抓落实，对每一项重点工作都细化方案，明确时间表、路线图、责任人。坚持转变作风抓落实，在市政府机关开展推进干部作风转变大讨论活动，查摆问题，狠抓整改。坚持督考合一抓落实，实施“回头看”，综合运用社会、媒体等监督方式，每一项任务都做到有部署、有检查、有评估、有奖惩，精准落实、有效落实正在成为每一名机关干部的习惯和自觉。

各位代表，五年砥砺前行，成绩来之不易。这是省委、省政府和市委正确领导的结果，是市人大、市政协和社会各界监督支持的结果，是全市上下团结拼搏的结果。在此，我代表市政府，向全市广大干部群众，向各位投资者和企业家，向各级人大代表、政协委员、各民主党派、工商联、无党派人士、人民团体，向离退休老领导、老同志，向中省直单位、驻营部队、武警官兵，向所有关心、支持营口发展的各界人士表示衷心感谢，并致以崇高敬意！

成就值得肯定，问题更需正视。我们要看到，受经济下行、做实经济数据等因素影响，过去五年我市地区生产总值、固定资产投资等指标没有完成预定目标任务；发展不充分、不平衡问题还没有很好解决，经济总量仍然较小，项目储备不足，城乡发展还不协调、不均衡；发展方式依然比较粗放，产业结构仍需优化，创新驱动尚未成为发展的主动力；生产要素配置的有效性还需提高，土地、资金、人才问题没有得到很好解决；文教卫生、社会保障等基本公共服务供给不足，生态环保和节能减排任务艰巨；偿还债务、弥补养老金缺口等刚性支出压力较大，财政收支矛盾突出；少数部门和个别干部作风不实、为官不为，政府自身建设任重道远。对此，我们将以久久为功的韧劲，逐步加以解决。

二、今后五年主要目标任务

各位代表，未来五年是全面建成小康社会的决胜期，也是新一轮老工业基地全面振兴的关键期。政府工作的总体要求是：全面贯彻党的十九大精神，以习近平新时代中国特色社会主义思想为指导，在省委、省政府和市委的坚强领导下，坚持稳中求进工作总基调，紧紧围绕“五位一体”总体布局和“四个全面”战略布局，按照高质量发展和“四个着力”“三个推进”要求，以供给侧结构性改革为主线，以做强港口、做足开放、做大民营为重点，优化营商环境，狠抓项目建设，力推改革创新，加速结构调整，保障和改善民生，打好三大攻坚战，促进全市经济社会持续健康发展，确保全面建成小康社会，争当辽宁振兴发展的先行者。

全市经济社会发展的奋斗目标为：到2020年，高质量完成“十三五”规划各项目标任务，全面建成小康社会；到2022年，经济社会持续平稳健康发展，每年主要指标增速均高于全国平均水平一个百分点，走在全省振兴发展前列。

——创新驱动更加有力。坚持把创新摆在全面振兴的核心位置，发展壮大新产业新业态新技术新模式，积极营造大众创业万众创新氛围，让创业创新无处不在、蔚然成风。到2022年，全社会研发经费投入占地区生产总值的比重超过全省平均水平，建成具有较强影响力的区域创新基地。

——开放优势更加凸显。深度融入“一带一路”，集聚开放要素，推动高水平双向开放，加快打造国际一流的营商环境，形成海港、空港、陆港、信息港多港联动，自贸区、开发区、园区、综保区多点支撑的开放型经济体系，使营口成为“一带一路”重要支点城市。

——城市发展更加协调。坚持城市带动农村、西部带动东部，加快形成资源共享、优势互补、布局合理、特色鲜明的城乡发展新格局。坚持把转方式、调结构放在更加突出的位置，着力推动产业转型升级，优化产业结构。到2022年，常住人口城镇化率达到70%以上。

——人居环境更加优美。坚持绿色发展理念，坚守生态底线，铁腕治理环境污染，大幅提高林木绿化率，加快形成绿色发展的生产方式、生活方式。不断完善城市功能，创建成为国家卫生城市、生态文明城市，把营口打造成为生态宜居、山海宜游、现代宜业的魅力城市。

——人民生活更加幸福。坚持人民至上、民生优先，扩大优质公共服务供给，织密织牢民生保障网，大力提升人民群众生活品质和保障标准，努力实现学有优教、病有良医、劳有多得、住有安居、老有善养、弱有强扶、闲有所乐，创建成为全国文明城市、国家食品安全示范城市。

破解发展难题，实现规划目标，必须放大优势功效，优化资源整合，兼顾均衡协调，奋力做好“五篇文章”。

突出港口牵动。依托亚欧大陆桥优势，支持港口拓展综合性港航服务，积极参与“一带一路”建设，打造区域性国际物流中心和国际海铁联运大通道重要枢纽。大力发展临港经济，以全域产业支撑港口发展，以港口发展带动全域经济，开创港产城联动发展新格局。

力推沿海先行。突出沿海经济带拉动全域发展龙头作用，利用国内、国外市场和资源，着力拓展发展空间。统筹推进重点园区建设发展，整合资源、优化布局、明确定位、完善机制，在扩大开放、创新发展、深化改革等方面走在全市前列，努力打造全市开放创新试验区、产业发展先行区。

坚持自贸区引领。完善体制机制，促进自贸区、综保区、高新区融合发展，持续推进政策制度的创新、系统集成和全面推广，综保区实现满负荷运转。大力发展高新技术产业和创业创新平台，把自贸区建设成为引领全市改革开放、创新发展的旗舰。发挥“三区叠加”的制度创新、对外开放、科技引领优势，吸引资本、人才、技术向营口集聚，吸引高新技术产业、加工贸易、金融、大宗商品交易等业态向自贸区集聚，自贸区力争5年新增注册企业突破1.5万户，运营率40%以上，进出口总额、实际利用外资、一般公共预算收入等主要指标实现“翻两番”。

强化民营支撑。毫不动摇鼓励、支持、引导民营经济发展，以企业需求为导向深化民营经济发展改革示范工作，加快形成民营经济科学发展的政务环境、市场环境、创新环境。大力培育民营企业家队伍，积极引导民营企业改制上市，不断壮大民营经济。民营企业增加值年均增长10%以上，占比达到80%以上。

加快东部发展。按照产业兴旺、生态宜居、乡风文明、治理有效、生活富裕的总要求，实施乡村振兴战略，开展东部发展攻坚行动。突出新型城镇化支撑，以城带乡，逐步完善东部地区基础设施，夯实发展基础；突出以产促农，鼓励生产要素向东部地区流动，建设新型农业经营体系，推动一二三产融合发展；突出公共服务均等化，着力提升东部地区教育、医疗、文化等事业发展质量和水平。东部地区农村常住居民人均可支配收入年均增加1000元以上。

各位代表：新蓝图已绘就，还需扬鞭策马。我们一定要坚定信心，担当作为，一定要拉高标杆，力争上游，为辽宁全面建成小康社会、实现新一轮全面振兴贡献更多的营口元素、营口经验和营口实践！

三、2018年工作安排

2018年是迈进新时代、全面贯彻落实党的十九大精神的开局之年，是决胜全面建成小康社会、实施“十三五”规划承上启下的关键之年，做好各项工作意义重大。全市经济社会发展主要预期目标为：地区生产总值增长7.5%，固定资产投资增长12%，一般公共预算收入增长8%，社会消费品零售总额增长8%，城乡居民收入增长与经济发展基本同步。

围绕上述目标，我们要抓好八项重点工作：

（一）强化项目支撑，推动沿海、县域经济实现新发展

突出项目中心地位，推进以园区为重点的沿海经济带建设，大力发展县域经济，着力提升全域整体发展水平。

狠抓项目建设。完善和落实招商引资优惠政策、奖励政策，突出主导产业，实行内外资并重、引资引智引技并举，通过委托招商、以商招商、对口合作等方式，瞄准欧美、日韩和国内重点地区，大力实施产业链招商、产业集群招商、龙头企业招商、临港经济招商，提高招商实效。清理盘活闲置资源，实施再包装、再招商、再利用。全年实际利用外资2亿美元，国内实际到位资金235亿元。强化项目建设分类调度和要素保障，实行挂图作战，压茬推进。全年开复工千万元以上项目465个，完成固定资产投资520亿元。

推进园区创新发展。围绕体制机制创新和产业发展，着力提升园区发展质量效益。各园区在明晰产业定位基础上，集中力量延长补齐产业链，做大做强主导产业。加快推进园区改革，对区位相邻、小而散和产业趋同的园区进行整合，全面推行公司化运作模式和全员竞聘管理。以重点园区为依托，推动海洋经济示范区申报建设，培育经济新增长点。重点园区一般公共预算收入、固定资产投资等主要经济指标至少要实现两位数以上增长。

补齐县域经济短板。围绕稳农业、强工业，大力实施“县域经济发展三年行动计划”，每个县都要建设一个工业产业园区、一个特色农产品深加工集聚区和一个服务业集聚区。实施乡村振兴战略，健全激励约束机制，激发县乡村三级抓发展的积极性。发展壮大村集体经济，选择40个行政村开展扶持村集体经济发展孵化工程。提升小城镇公共服务水平和承载能力，引导农村人口就地就近向城镇集中。重点加快盖州北海海蚀风情、大石桥建一黄丫口等特色小镇建设，推动高坎镇等创建成为省级特色乡镇。

（二）依托港口优势，构建现代化产业新体系

围绕港口核心资源，推动传统产业转型升级、新兴产业快速发展，努力建设现代化产业体系。

集聚发展临港产业。把握辽宁港口整合契机，编制港城一体化发展规划，打造全域临港产业区。推进与港口密切相关的港航物流业、临港大工业等现代临港产业集聚发展，加快中粮集团、和润集团粮油深加工等项目建设，强化配套产业引进，打造主业突出、特色鲜明、链条完整、支撑有力的临港产业集群，促进产业落地、货值落地、税收落地。

着力发展实体经济。树立大抓实体、大兴实业鲜明导向，以更有力的举措推动互联网、大数据、人工智能与实体经济深度融合，鼓励实体经济企业专注主业，加强品牌建设，增强核心竞争力。做强先进装备制造、快速消费品、精细化工、钢铁及深加工四个主导产业，做优镁质材料、汽保、乐器三个特色产业，做大新材料、新能源两个战略性新兴产业。加快实施智能制造工程，推进1个国家级、11个省级和50个市级示范项目建设。加大企业帮扶力度，解决企业运行和项目建设中遇到的问题，着力推进现有企业扩大再生产。实施镁产业提标升级，促进低端产业和落后产能有序退出。落实各项减税降费优惠政策，推动更多企业参与电力直接交易，进一步降低企业成本。全年规模以上工业增加值增长12%。加快建筑业转型发展，支持本土建筑企业做大规模、做强实力。

加快发展现代服务业。围绕港口、装备制造业加快发展生产性服务业。提升物流业发展水平，加快构建冷链物流体系，支持大型物流中心、配送中心、分拨中心发展，集中力量打造一批规模化、智能化物流企业。促进金融业助力实体经济发展，全年新增金融机构5家以上，挂牌上市企业5家，社会融资总量超过550亿元。优化生活性服务业结构，培育健康养老、家政服务等业态，实施“互联网+流通”行动计划，加快实体零售业转型升级，发展社区连锁便利店，提升便民服务水平。围绕打造支柱产业，整合滨海、温泉、沟域生态和历史文化等特色资源，加快旅游项目建设和宣传推介，构建“一带两核四组团”大旅游格局，旅游总收入增长12%。综合施策推进房地产去库存，加大公租房保障力度，商品房销售面积增长10%。全年服务业增加值增长7.5%。

大力发展现代农业。全年粮食产量稳定在68万吨左右，调减玉米种植面积1万亩。立足营口优势农业资源，以“三品一标”品牌建设为抓手，着力推进“一镇一业”“一村一品”，打造特色农业、精品农业、休闲农业。全年新建续建千万元以上农业产业化项目18个，创建标准示范园6个，新增“三品”认证面积6万亩，新增农民专业合作社100家。稳步推进农村土地“三权”分置改革，全面完成农村土地确权登记颁证任务，激发农业发展活力。全年农业增加值增长3.5%。

（三）发挥自贸区引领带动作用，集聚全面开放新优势

深度融入“一带一路”，以更宽更广的视野布局对内对外开放，推动营口开放发展向更深层次、更高水平迈进。

加快自贸区突破发展。加速政策创新和特色案例培育推广，推进证照分离、证照联办改革，建设国际贸易“单一窗口”子网站，引领全市“放管服”改革。中央商务区全面投入运营，加快实施自贸区至营口港铁路专用线工程，综保区实现封关运营。着力推进项目建设和产业发展，新增注册企业超过3000户，财政收入增长100%。

提升外经贸发展水平。坚持稳份额、调结构、增效益，强化对现有出口企业的扶持服务，促进外贸出口稳定增长。进一步扩大先进技术、重要装备和零部件、紧缺资源性产品进口，助力产业升级发展。参与建设全省“一带一路”综合试验区，以俄罗斯、中东欧、东南亚以及非洲国家为重点区域，引导企业开展国际产能合作，带动装备、技术、服务“走出去”，确保进出口总额占全省的份额稳中有升。

深化区域对接合作。加强与连云港、温州、佛山、泉州等地区的对接合作，形成一个领导、一个部门、一个园区、一个城市合作模式，共建一批合作示范园区或“飞地经济”“异地园区”，使营口成为南方先进地区产业转移的示范区。

（四）加大改革力度，激发体制机制新活力

把改革作为推动振兴发展的根本路径，以供给侧结构性改革为主线，全面深化相关领域改革，完善体制机制，破除无效供给，释放错配资源，激发市场活力。

深入实施“放管服”改革。围绕职权下放不到位问题，加大简政放权力度，深入推行市、县行政审批一体化改革，赋予园区更大范围的经济管理权限，确保“放到位”。创新监管模式，落实监管责任，健全审管互动机制，强化事中事后监管，着力解决审、管分离后管理弱化问题，确保“管得住”。依托“互联网+政务服务”改革，加快建成全市审批、管理、服务“一张网”，推动乡镇（街道）、村（社区）等基层服务网点与网上服务平台有效对接，努力做到企业市场准入“全网通办”、居民社区事务“全市通办”、政府政务信息“全域共享”，确保“服好务”。

统筹推进重点领域改革。深化国资国企改革，加快市城投公司转型，稳步建设国有资本运营体系。全面推进国有企业混合所有制改革，继续推进事业单位转企组建集团工作，探索创新公益类事业单位人员编制管理，提升事业编制资源使用效益。完善预算管理体制改革，实行全口径预算管理，健全重点支出保障机制，优先安排保工资、保养老金、保运转等刚性支出。深化县乡财政体制改革，理顺分配关系，从今年开始5年内乡镇新增财力全部留给乡镇，增加乡镇可支配收入，增强乡镇自我发展能力。实施投融资体制改革，发挥政府投资引导作用，依法有序推广PPP模式，激发民间投资活力，民间投资占比达到80%以上。

持续优化营商环境。以企业需求为导向，有的放矢，不断改进企业投资经营的制度环境。强化“店小二”意识，发扬“五皮”精神，做好“妈妈式”服务，坚决摒弃“官本位”思维。深入落实项目和企业全周期“四个一”帮扶制度，及时解决问题，维护企业合法权益，把外面的企业招进来，把本地的企业发展好。加大明察暗访、社会监督力度，让市场主体和服务对象提意见，严肃查处“庸、懒、散、慢、乱”等不良风气以及托关系、“走后门”等不正当行为，发现一起查处一起，绝不姑息。从政府部门和工作人员做起，以诚信政府、法治政府建设为引领，构建诚信社会、法治社会。

做大做强民营经济。推进民营经济发展改革试点建设，营造更加适宜民营经济发展的“营口气候”。继续实施“个转企”“小升规”“大做强”工程，全年完成“个转企”800户，新增规模以上企业50户。推进龙头企业和有潜质的中小企业加快建立现代企业制度，提高经营管理水平。更加关注关爱企业家，依法保护企业家产权，在资本运作、技术攻关、人才引进等方面为民营企业排忧解难。弘扬企业家精神，营造尊重、激励和保护企业家干事创业的社会氛围，打造现代企业家营口军团。

（五）加快科技创新，培育发展新动能

坚持把创新作为引领发展第一动力，大力提升创新能力和活力，广聚创新资源，在创新中赢发展、赢未来。

建设重大创新平台。推动高新区“一区多园”联动发展，争创国家创新型特色园区，力争进入全国高新区100强。建立营口市技术交易信息、研发检测等公共服务平台，转化科技成果60项，培育省、市工程技术研究中心10家。推进营口众创空间、九州创客等创业创新平台高端化发展。

优化创新生态。实施高新技术企业三年倍增计划，新增国家级高新技术企业30家。推进国家知识产权试点建设，全年有效发明专利增长10%以上。建立健全创新发展的体制机制，落实科技创新扶持政策，深化产学研合作，推动科技与金融深度融合，构建创新服务体系，营造崇尚创新、鼓励创新的良好环境。全社会研发经费投入增长15%以上。

集聚创新人才。强化创业创新服务，吸引外出人才回流营口，启动实施万名高校毕业生引进计划。强化校企合作，大力推广订单式人才培养模式。实施技能人才名师、名匠、名校工程，培养“营口工匠”和“首席工人”。

（六）加强城乡建设，构筑区域协调发展新格局

统筹推进新老城区建设，加快完善城市功能，填平补齐薄弱环节，实现区域共同发展。

促进城际协同发展。主动接轨环渤海经济圈，加强与其它城市的战略合作。依托辽宁沿海经济带主轴城市和沈阳经济区出海口优势，加强与周边城市的沟通协调，创新合作机制，实现基础设施互联互通、生态环境联防联治、产业发展协同协作。

优化市域空间布局。树立大统筹理念，积极推进“多规合一”，逐步形成覆盖全市的“一张蓝图”。加快推进北部城区和大石桥、盖州和北海、鲅鱼圈和仙人岛三轴一体化发展，加快构筑以中心镇、重点镇和特色小镇为依托的城镇体系，形成一带三轴、多点支撑的城市发展格局。

完善基础设施。优化资源配置，加快实施营口职业技术学院新校区等项目建设，进一步完善营东新城、沿海产业基地功能，提升人气。逐步改造老城区，推进文化综合体、营口火车站改造升级等项目建设，新建聪慧路、清华路部分路段，改造新兴大街、东升路等街路道板、路灯，实施供水管网改造、主城区公厕改造等工程，启动无害化、资源化垃圾处理厂建设。加强乡村道路等硬件设施建设，改善农村生产生活条件。

实施精细化管理。推进智慧营口建设，构建智慧城市应用体系，打造以人为本、惠及全民的民生服务新模式。对市区主次街道实施分类管理，集中整治违法建设、违规占道经营等行为，营造整洁、美观、舒适、有序的市容市貌。优化农村人居环境，完成120个村环境综合整治，实施美丽乡村示范村工程19个。做好国家森林城市复检工作，争创全国文明城市、国家卫生城市。

（七）打好三大攻坚战，实现补短板新突破

围绕补齐全面建成小康社会短板，统筹规划、有序推进，坚决打好防范化解重大风险、精准脱贫、污染防治攻坚战。

积极防范化解各类风险。实施政府债务限额管理，严控新增债务规模，通过处置闲置资产、债务置换等途径，积极化减存量债务，有效防范政府债务风险。采取强化征缴管理、预算安排等举措积极筹集资金，严格规范基金支出，保证养老金及时足额发放。强化部门联动，建立健全企业金融风险预警机制，监控掌握企业纳税缴费情况，及时处理解决企业风险隐患。

加大精准脱贫力度。坚持产业扶贫为先，实现贫困户产业带动全覆盖。加大教育扶贫投入，阻断贫困代际传递。实施健康扶贫工程，防止因病致贫、返贫。强化社保兜底扶贫，为全市建档立卡贫困人口购买扶贫救助保险。全年完成危房改造400户，脱贫7000人，贫困村退出10个。

强化污染防治。全面做好大气、水、土壤污染防治工作，打赢蓝天保卫战，完成拆除小锅炉、淘汰黄标车及老旧车辆等任务，实现PM2.5和PM10浓度“双下降”。加快城镇污水处理厂提标改造进程，完成北部污水处理厂建设，实现主城区污水全部收集处理、达标排放。加强黑臭水体及入海河流整治，确保年底前沙河、熊岳河水质达标；加强饮用水源地保护，确保饮水安全。开展污染源详查工作，加大垃圾分类试点工作力度，加快生活垃圾处置设施建设，安全规范处置各种生产垃圾，减少土地污染。强化排污者责任，完善生态补偿机制。加大执法、监测力度，严厉打击破坏生态环境的各类违法行为。

（八）推进民生改善，顺应人民群众新需求

坚持以人民为中心的发展思想，加大民生投入，办好21件重点民生实事。

加大社会保障力度。坚持就业优先，全年城镇新增实名制就业2万人，城镇登记失业率控制在3%左右。实施全民参保计划，实现法定人员保险全覆盖。继续提高保障标准，最低生活保障标准平均提高5%以上，适当提高企业退休人员基本养老金、城镇居民医保政府补助、新农合政府补助等标准。统筹实施医疗救助、慈善救助、残疾人补贴、特困职工补助等精准救助，有效保障困难群众基本生活。完善城镇医疗保险制度，全面实行市级统筹“五统一”。健全城乡养老服务体系，鼓励社会力量参与养老服务，提高服务质量。实行社区共建共治共享，提升社区建设、管理、服务水平。

加快发展社会事业。坚持教育优先发展，优化校长、教师交流轮岗培训机制，着力解决“择校热”“大班额”等突出问题。加强学前教育，推进义务教育优质均衡发展，大力提升普通高中教育质量，支持营口职业技术学院建设高水平现代化职业院校，推动营口理工学院建设应用型大学。强化职业教育和特殊教育，鼓励民办教育、远程教育发展，培育特色教育品牌。深化医药卫生体制改革，推进医联体建设，完善基层医疗卫生服务体系，建设健康营口。实施文体惠民工程，完成乡镇、村级基层综合性文化服务中心年度建设任务，建设改造100个农村文化（体育）示范广场，推进专业博物馆、纪念馆建设。整合文化体育资源，打造鲅鱼圈马拉松等精品赛事。举办全民体育休闲运动会，提升群众健身意识和健康水平。

创新社会治理。深入开展“七五”普法，增强法律服务能力和水平，提高全社会法治素养和道德素质。深化立体化社会治安防控体系建设，依法打击严重暴力和影响群众安全感的违法犯罪活动，努力建设更高水平的平安营口。健全多元化矛盾纠纷化解机制，妥善处理各类信访问题。牢固树立安全发展理念，严格落实安全生产责任，坚决遏制重特大安全生产事故发生。实施食品安全战略，完善食品药品安全监管治理体系，积极创建国家食品安全示范城市。

认真做好国防教育、民兵预备役和人民防空工作，巩固提高“双拥”工作成果，促进军民融合发展。加强国家安全、统计、民族、宗教、史志、档案、气象、地震等工作。推动慈善、红十字、老龄、妇女儿童、残疾人等各项事业再上新台阶。

四、努力建设人民满意政府

打铁必须自身硬。我们要按照市委的统一部署和要求，切实加强政府自身建设，以永不懈怠的精神状态和一往无前的奋斗姿态，勇于担当、主动作为，履行好党和人民赋予的神圣职责。

坚持以学强政。深入学习贯彻党的十九大精神，牢固树立“四个意识”、坚定“四个自信”，做到对党和人民绝对忠诚。树立终身学习理念，注重培养专业能力、专业精神，切实解决“知识恐慌”“本领恐慌”问题。坚持理论联系实际，深入开展调查研究，切实把理论知识、上级政策举措与营口实际紧密结合，提升履职能力和工作水平。

坚持为民执政。把人民利益摆在至高无上的地位，尽心竭力为群众解难题、办实事、增福祉。坚持主动服务、靠前服务、高效服务，认真听取群众意见，注重解决实际问题。畅通互动渠道，及时解决企业和群众诉求，打通服务企业、联系群众的“最后一公里”。

坚持依法行政。提高运用法治思维和法治方式深化改革、推动发展的能力，把政府工作全面纳入法治轨道。认真落实市人大及其常委会的决议、决定，主动接受市人大及其常委会的法律监督、工作监督和市政协的民主监督，办理并落实好各项建议、提案，不断改进提升政府工作。深入推进政务公开，及时回应社会关切，让权力在阳光下运行。

坚持务实理政。始终把抓落实作为政府工作的生命线，以“干”字当头、“实”字托底，认真履职尽责、真抓实干，切实做到真心落实、迅速落实、精准落实、有效落实。充分发扬钉钉子精神，锲而不舍、步步为营、久久为功，切实做到较真章见好效。坚决不做表面文章、不搞短期行为、不玩击鼓传花、不当甩手掌柜，坚决反对口号空喊、文件空转、人力空耗、工作空白。建立健全激励约束机制，为担当者担当、为干事者铺路，让层层负责、人人担责成为自觉、成为常态。推进政府管理精细化，将细致入微的理念融入工作全过程，做到紧之又紧、细之又细、实之又实。

坚持廉洁从政。严格落实全面从严治党各项要求，抓好党风廉政建设和反腐败工作，时刻把党的纪律和规矩挺在前面，按党性原则办事、按政策法规办事、按制度程序办事。强化审计监督，严管公共资金使用、公共资源交易等关键领域和关键环节。全面落实“一岗双责”，坚决贯彻中央八项规定精神和实施细则，一以贯之查找和纠治“四风”问题，努力营造干部清正、政府清廉、政治清明的良好局面。

各位代表：使命重在担当，实干铸就辉煌。让我们更加紧密地团结在以习近平同志为核心的党中央周围，在省委、省政府和市委的正确领导下，不忘初心，牢记使命，开拓进取，攻坚克难，为决胜全面建成小康社会、实现老工业基地全面振兴、建设富强民主文明和谐美丽的新营口而努力奋斗！
